# Detecting Specific Health-Related Events Using an Integrated Sensor System for Vital Sign Monitoring

**DOI:** 10.3390/s90906897

**Published:** 2009-09-01

**Authors:** Mourad Adnane, Zhongwei Jiang, Samjin Choi, Hoyoung Jang

**Affiliations:** 1 Department of Mechanical Engineering, Faculty of Engineering, Yamaguchi University, 2-16-1, Tokiwadai, Ube, Yamaguchi, 755-8611, Japan; E-Mails: adnanemourad@yahoo.fr (M.A.); m013ve@yamaguchi-u.ac.jp (H.J.); 2 Department of Biomedical Engineering, School of Medicine, Kyunghee University, #1 Hoeki-dong, Dongdaemun-gu, Seoul 130-702, Korea; E-Mail: samdoree2@hotmail.com (S.C.); 3 Healthcare Industry Research Institute, Kyung Hee University, Seoul, 130-702, Korea

**Keywords:** wearable sensor, home healthcare, integrated sensors system, RR interval, respiratory cycle variability, time series analysis, sleep apnea

## Abstract

In this paper, a new method for the detection of apnea/hypopnea periods in physiological data is presented. The method is based on the intelligent combination of an integrated sensor system for long-time cardiorespiratory signal monitoring and dedicated signal-processing packages. Integrated sensors are a PVDF film and conductive fabric sheets. The signal processing package includes dedicated respiratory cycle (RC) and QRS complex detection algorithms and a new method using the respiratory cycle variability (RCV) for detecting apnea/hypopnea periods in physiological data. Results show that our method is suitable for online analysis of long time series data.

## 1. Introduction

The continuous changes in life quality over the last decades have led to new daily life needs. For instance, the increase in life expectancy and population ageing has resulted in an increase in the number of applications of new technologies to support elderly people. An important illustration of this is the growing field of in-home health care devices. This tendency is expected to be maintained in the near future since the speed of ageing is likely to increase over the coming decades [1]. On the other hand, cardiovascular and cardiorespiratory diseases are constantly growing among world population. Many reasons explain this, among them the mental stress caused by the modern life, which is full of deadlines, expectations and disappointments. Actually, cardiovascular diseases represent the first cause of death (29.34% of causes) worldwide while the cardiorespiratory diseases represent 6.49% of the death causes according to the World Health Organization (WHO) [2].

Even though the importance of the early diagnostic of such diseases is obvious, some cardiovascular and cardiorespiratory-related illnesses remain under-diagnosed. Sleep related diseases are a typical example of these illnesses. Indeed, sleep diseases remain extremely under-diagnosed, in spite of their high impact on public health and this is mainly caused by the lack of proper diagnosis tools [3]. Currently the conventional system for sleep study, polysomnography (PSG), is constrictive and expensive. In fact, PSG is used in exclusively hospital environments. Therefore, the patients are required to stay in hospital for a whole night. It is therefore clear that there is an unmet demand for development of in-home systems for monitoring cardiorespiratory disorders and the study of sleep conditions [4–6]. Such systems can be of a great help for physicians in diagnosis and can help to solve bottleneck problems in hospitals. Along with this, an integrated sensor system for the acquisition of electrocardiography (ECG) and respiratory signals was developed in our laboratory [7]. The sensors used are a polyvinylidene fluoride (PVDF) film and conductive fabric sheets integrated into a wearable belt. Specific hardware was developed for acquiring ECG and respiration signals using the cardiorespiratory belt sensor. In addition, signal processing algorithms specifically designed for this system were developed [8,9]. Noticeably, the signals acquired with this system are quite different from the conventional ones. As an illustration of this, the ECG acquired by the PVDF sensor is actually a heartbeats signal. The difference is subtle but has important consequences. The PVDF has piezoelectric properties. These properties are used in our system for detecting the movement in the surface of the body corresponding to heartbeats. Therefore, motion artifacts are more important in the heartbeats signal acquired with the PVDF sensor. Conversely, the conductive fabric sensor detects the electrical potential on the surface of the body like conventional three lead sensors. However, the base wander is much higher in the conductive fabrics sensor. The reason for is that this sensor is not firmly attached to the body in the manner the electrodes are attached with patches. These noises require specific signal processing, as mentioned earlier, in order to extract valuable information.

In order to make a reliable system for in-home healthcare monitoring a system should include three main points. First, it should properly detect physiological signals, in our case ECG and respiration signals. Second, the detected signals should be processed to get valuable information. Third, the obtained information should be used in particular methods for detecting specific health-related events; this point represents the application of the system. The two first points were already discussed elsewhere. However, we will mention briefly in this paper the main parts of these two points, i.e., sensor system design, the acquisition system and the applied signal processing. The third point, which is about the application of the in-home healthcare system, represents the main theme of this paper.

The cardiorespiratory belt sensor was used for the acquisition of data during night (sleep study). In order to evaluate these data it is important to develop a method for the detection of the health-related events within it. In our research, we used the respiration signal and the ECG signal as the basis of our processing. Actually, the changes in the cardiorespiratory signals are strongly correlated with health-related events occurrences. Indeed, several researchers have used cardiorespiratory information for the detection of specific events such as, automated apnea detection system [3], mental stress evaluation [10–12] and sleep studies [13–17]. Therefore, in this paper we present a new method for apnea/hypopnea event detection based on processing of respiration data. This method is based on the use of the respiratory cycle variability (RCV) in the same manner the heart rate variability (HRV) is used. Indeed, the respiratory signal is derived from the raw signal acquired using the PVDF sensor using a special function. This function is used to obtain good quality respiration signal, which is based on the calculation of local energy of the raw signal obtained with the PVDF sensor. Then a power spectrum density estimation of the obtained signal is done. The LF/HF ratio corresponding to the power in the low frequency over the power in the high frequency of the PSD estimation is calculated. The low frequency power (LF) corresponds to abnormal respiratory frequencies, whereas the high frequency (HF) power corresponds to normal respiration frequencies. The apnea/hypopnea events are easily detected by a peak appearing in the plot of the variations of LF/HF versus time. A dataset of sleep signals measured using the cardiorespiratory belt sensor was used for validating this method. Experiments showed that it is possible to use our system which is composed of the cardiorespiratory belt sensor and dedicated signal-processing package for the detection and the evaluation of some specific health-related events, for instance apnea/hypopnea episodes.

This paper is organized as follows: in Section 2, the proposed integrated sensor system is described. In Section 3, the data processing techniques developed for this system are explained, with a focus on the new method we recently developed. In Section 4, experiments using the cardiorespiratory belt sensor for sleep studies are described. In Section 5, results obtained using the proposed method applied to the sleep data are shown. A discussion is made in the sixth section and we finish with a conclusion.

## 2. Proposed System

**Figure 1.**
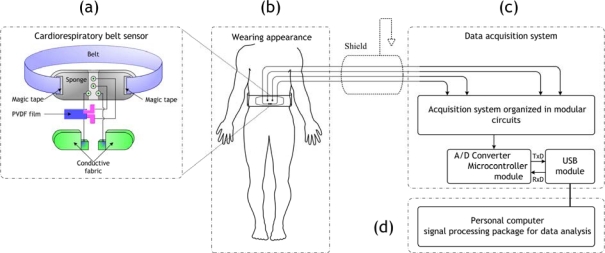
Overview of the cardiorespiratory belt sensor system. (a) Cardiorespiratory belt sensor. (b) Wearing appearance of the cardiorespiratory belt sensor. (c) Hardware system for signals acquisition. (d) Data processing techniques within personal computer.

Figure 1 depicts an overview of the cardiorespiratory belt sensor system. This system consists of a belt type sensor probe, data acquisition and communication devices, and dedicated signal processing package to extract the cardiorespiratory information. The sensors and hardware systems are described in the following subsections. The signal processing techniques are treated in a separate section.

### 2.1. Integrated Sensors System

The belt sensor probe made in our laboratory is composed of a couple of conductive fabric sheets and a polyvinylidene fluoride (PVDF) film. The PVDF film is used to detect both the ECG and the respiratory signals and the conductive fabric sheets are used to detect the ECG. In the following a detailed description of each sensor is given.

#### 2.1.1. PVDF Film Sensor

**Figure 2.**
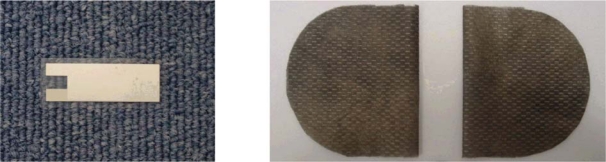
PVDF film sample (left panel) and conductive fabric sheets sample (right panel).

The PVDF film is a piezoelectric polymer, which is very sensitive towards changes in the strain applied on it. The PVDF film used in our system (left panel in Figure 2), with dimensions 16.5 mm × 37 mm × 0.003 mm, was used in the belt-type sensor in order to measure the respiratory cycles corresponding to the abdomen rising and falling movements, while it can also detect heartbeats corresponding to small movements in the surface of the body.

#### 2.1.2. Conductive Fabric Sensor

The sensor used for ECG detection is composed of two sheets of copper coated conductive fabric as shown in Figure 2 (right panel). These two sheets were interlaced within the belt sensor probe to measure the ECG signal. The characteristics of the conductive fabric sheets are 3 × 10^−3^ mm in thickness, 9.168 × 10^−3^ kg/m^2^ in weight and 49.215 × 10^3^ kg/m^2^ in strength.

### 2.2. Hardware System

**Figure 3.**
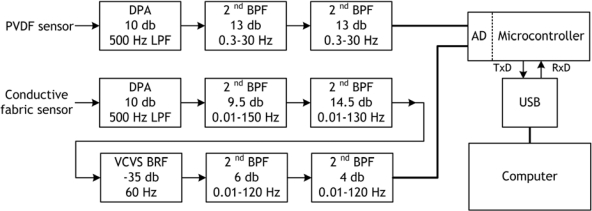
Block diagram of the signals acquisition circuits for PVDF film and conductive fabric sensors.

A hardware system was built for each sensor in order to correctly acquire the ECG and the respiratory signals using the belt sensor. A modular system was adopted, where three basic high quality and flexible hardware modules: a differential pre-amplifier (DPA) module with 500 Hz low-pass filter (LPF), high quality band-pass filter (BPF) modules and voltage controlled voltage source (VCVS) band-rejection filter (BRF) module, were designed and assembled together in the hardware circuit. The block diagram of the acquisition circuits for PVDF and conductive fabric sensors is shown in Figure 3.

### 2.3. Cardiorespiratory Belt Sensor System

**Figure 4.**
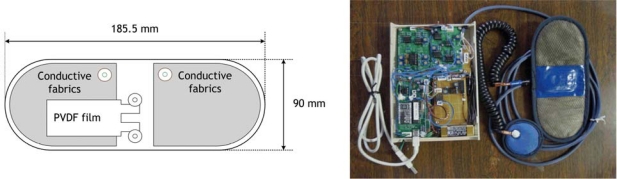
Schematic of the belt probe (left panel) and photograph of the cardiorespiratory belt sensor with the hardware for signals acquisition (right panel).

**Figure 5.**
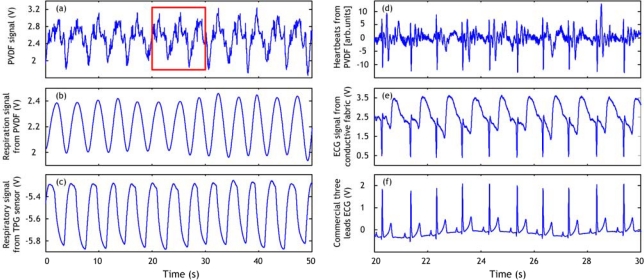
(a) Signal acquired using PVDF sensor. (b) Respiration signal derived from (a) by applying LPF (0.33 Hz cutoff frequency, −61 db Blackman window). (c) Respiration signal obtained with TPG sensor. (d) Heartbeats derived form (a), in the rectangle range; by applying derivative function and HPF (1 Hz cutoff frequency, −61 db Blackman window). (e) ECG signal obtained with conductive fabric sensor in the same range of (d). (f) ECG signal obtained with commercial three leads ECG in the same range of (d, e).

The PVDF film and conductive fabric sheets are knitted in the belt probe as depicted in the schematic of Figure 4 (left panel). The actual cardiorespiratory belt type sensor and the hardware for the signal acquisition are shown together in Figure 4 (right panel). The cardiorespiratory belt sensor should be worn around the waist. An example of the ECG and respiration signals acquired using the cardiorespiratory belt sensor is depicted in Figure 5, together with a commercial thermistor-type pneumography (TPG) sensor (for validating respiration signal acquisition) and commercial three lead ECG sensor (for validating ECG signal acquisition).

Figure 5 shows signals acquired using the cardiorespiratory belt sensor and the commercial systems. It is noticed that the respiration signal is easily derived from the signal obtained with the PVDF sensor. As remark, we can see that there is a slight delay between the respiration derived from PVDF sensor output and the TPG respiration signal. This is simply caused by the nature of each signal. Actually, the TPG detects the respiration inhalation and expiration through changes of the temperature whereas the PVDF detects the movements of rising and falling of the abdomen corresponding to the inhalation and expiration phases. In fact, these two aspects are delayed in time where the inhalation and expiration happens and then the thoracic muscles are contacted in an eccentric or concentric fashion. On the other hand, the ECG acquired by the conductive fabric sensor is of good quality and the different waves within it correspond exactly to the ones in the commercial three lead ECG signal. The heartbeats signal is derived from the signal acquired with the PVDF sensor quite well, as it is clear from Figure 5d; although ECG signal obtained with the conductive fabric sensor is of better quality. The signal processing techniques used for deriving respiration and heartbeats from the signal acquired with the PVDF sensor in this figure are simple. More powerful methods are presented in the next section.

## 3. Data Processing Techniques

The signals acquired with the cardiorespiratory belt sensor need special signal processing in order to extract the ECG and respiration signal information properly. In this section we present three techniques developed in our laboratory for extracting useful information from the cardiorespiratory belt sensor’s signals. Two techniques were already treated in previous publications and will be introduced briefly, whereas the third one, which is a new method, is explained in depth.

### 3.1. Respiratory Cycle (RC) Detection

Here the extraction of the respiratory cycle from the PVDF film sensor output is explained. Let *R* (*j*) be the obtained discrete signal; *j* represents discrete time. First the mean is removed from this signal:

(1) $$R_{M}\;(j)=R(j)-\bar{R}(j)$$

Then, it is normalized to the maximum value:

(2) $$R_{N}\;(j)=\frac{R_{M}\;(j)}{\mathrm{Max}(\left|R_{M}\;(j)\right|)}$$

**Figure 6.**
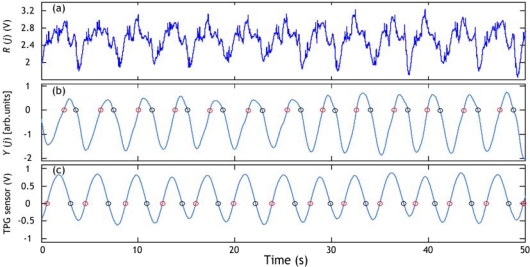
Extraction of respiratory cycle information. (a) PVDF sensor output. (b) Respiration signal derived from PVDF output. (c) Respiration signal obtained with TPG sensor. Circles represent respiratory cycle information estimated during inhalation time (positive slope) or respiratory cycle estimated during expiration time (negative slope). Arb.units expresses arbitrary units.

Then, wavelet decomposition with the A7 approximation (0–3.90625 Hz) is used to cut off the unwanted high frequency components and the A15 approximation (0–0.0153 Hz) is used for eliminating the baseline wander noise from *R_N_* (*j*). In addition, a second-order LPF with 0.3 Hz cut-off frequency is applied to the signal, reconstructed by the wavelet components of A7–A14, to extract the respiratory cycles. The obtained signal is defined as *Y* (*j*). Figure 6 shows an example of respiratory cycle information extraction from the PVDF sensor output (the same one of Figure 5a). The respiratory signal obtained with the TPG sensor is also used for estimating the respiratory cycle. A respiratory cycle is defined by the time elapsing between two successive circles on the positive slope or between two successive circles on the negative slope. For instance, if we decide to use the circles on the negative slope we find that there are twelve cycles in the example of Figure 6. The average respiratory cycle is estimated as 3.77 s with PVDF sensor results (Figure 6b) and 3.75 s with the TPG results (Figure 6c). The results are quasi similar.

### 3.2. RR Interval Calculation

The construction of the RR interval series is very important in a computer-based processing of the ECG signal. The RR intervals are basically calculated by detecting the main wave within the ECG signal: the QRS wave; in particular the R wave. The RR interval series are defined as the time elapsing between adjacent R peaks. We introduce, here, briefly the method we are using for RR interval series calculation.

**Figure 7.**
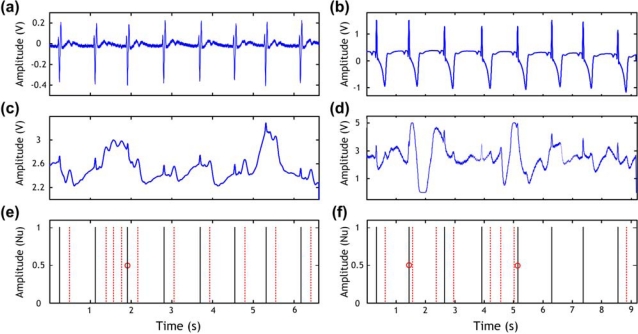
ECG detected by commercial three leads ECG device in (a) Example 1 and (b) Example 2. (c) Heartbeats detected by the PVDF sensor corresponding to Example 1 in (a). (d) ECG signal detected by the conductive fabric sensor corresponding to Example 2 in (b); (e) and (f) show the true peaks expressed by solid lines and misdetections expressed by dotted lines, and the missed peaks marked by circles corresponding to (c) and (d) respectively. Nu expresses normalized units.

The PVDF and conductive fabric sensors outputs are subject to intermittent strong noise episodes caused mainly by body movements. In order to get accurate R wave information we developed a QRS detection algorithm dedicated for the cardiorespiratory belt sensor. This algorithm is based on the combination of heart rate indicators and morphological ECG features (for details see [9]). Figure 7 shows the ECG signals obtained by both a commercial three lead ECG device in Figure 7a,b and our cardiorespiratory belt sensor in Figure 7c (PVDF sensor) and in Figure 7d (conductive fabric sensor). Figure 7e,f shows the peaks that should be detected (solid lines), the wrongly detected peaks (dotted lines), and the missed peaks (cycles). These possible detection errors are induced by the noisy signal. Short periods of bad quality signals induce false or missed peaks, as shown in Figure 7 if a conventional QRS detection algorithm is used. It is to be noticed that our algorithm succeed in detecting exactly the peaks in both examples (PVDF and conductive fabric sensors) which are indicated by the solid lines in Figure 7e,f).

**Figure 8.**
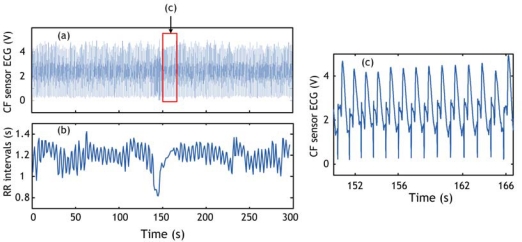
(a) ECG signal detected using conductive fabric sensor. (b) RR interval series calculated from (a). (c) Episode of ECG signal in the range of the rectangle shown in (a).

The RR interval series are then calculated from the output of the QRS detection algorithm. An example of RR series obtained using our cardiorespiratory belt sensor and QRS detection algorithm is shown in Figure 8.

### 3.3. Respiratory Cycle Variability (RCV)

Here we present a new and simple method for the detection of respiratory-related events in physiological data. This method is based on the use of the respiratory cycle variability for detecting apnea/hypopnea events. This method includes three steps.

First the raw signal acquired from PVDF sensor noted as *R*(*j*) is treated in a process where a local energy is calculated, this energy is estimated in windows of time; *j* represents discrete time. This has the effect of eliminating the noisy fluctuations and averaging the energy of the PVDF sensor output in the windows. If the window size is chosen sufficiently low the respiration signal will be clearly obtained, i.e., window size ≤ respiration period. In the following a mathematical definition of the energy estimation is given.

Suppose *r*(*t*) is a continuous signal in time then the energy *μ*(*t*,δ) at a given window width 2δ is defined as:

(3) $$\mu (t,\;\delta )=\frac{1}{2\delta }\;\int_{t-\delta }^{t+\delta }{\left(r(\tau )-\bar{r}(t)\right)}^{2}d\tau$$

(4) $$\text{With}:\;\;\bar{r}(t)=\frac{1}{2\delta }\int_{t-\delta }^{t+\delta }r(\tau )d\tau$$

where 2δ is the window size. We inject Equation (4) in Equation (3), then Equation (3) can be rewritten:

(5) $$\mu (t,\delta )=\frac{1}{2\delta }\int_{t-\delta }^{t+\delta }r{\left(\tau \right)}^{2}d\tau -\bar{r}{\left(t\right)}^{2}$$

It is clear that the local energy as defined in Equation (5) induces heavy computations in a computer program. Actually, for a given δ, (4δ + 1) summations and (2δ + 3) multiplications are needed to calculate the energy at time *t*.

Then simplification is introduced using two values:

(6) $$\mu_{1}\;(t)=\int_{-\infty }^{t}r(\tau )d\tau$$

(7) $$\mu_{2}\;(t)=\int_{-\infty }^{t}r{\left(\tau \right)}^{2}d\tau$$

Then Equation (5) can be rewritten as:

(8) $$\mu (t,\delta )=\frac{1}{2\delta }\left(\mu_{2}(t+\delta )-\mu_{2}(t-\delta )\right)-\frac{1}{{\left(2\delta \right)}^{2}}\;{\left(\mu_{1}(t+\delta )-\mu_{1}(t-\delta )\right)}^{2}$$

The simplification used in Equation (8) is based on recurrence principal; for example *μ*_1_(*t* + 1) = *μ*_1_(*t*) + *r*(*t* + 1) and *μ*_2_(*t* + 1) = *μ*_2_(*t*) + *r*(*t* + 1)^2^. Then for a given δ, only six summations and three multiplications are needed to calculate the energy at time *t*. It is noticed that the calculation time is independent on window parameter δ values. The energy noted *μ*(*j*,δ) represents the respiration signal derived from the PVDF sensor output, where the index *j* represents the time and *R*(*j*)^2^ is the signal used to calculate the energy (noted *r* (*t*) in Equations 1–5).

Second, a periodogram estimation of the power spectrum density (PSD) with Hamming window is obtained using the signal *μ*(*j*,δ).

**Figure 9.**
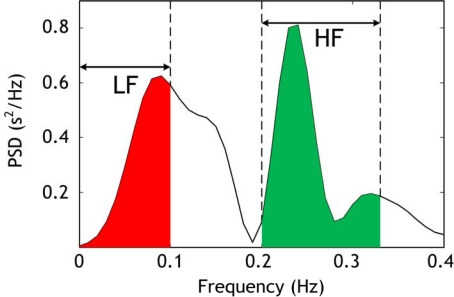
Power spectrum density (PSD) of 30-s length respiration signal corresponding to the record 1 of the sleep dataset. Low frequency (LF) is shown in red while high frequency (HF) is shown in green.

**Figure 10.**
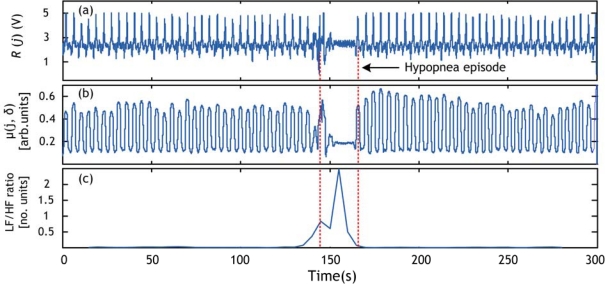
Hypopnea detection using respiratory cycle variability (RCV) for record 1. (a) PVDF sensor output. (b) Respiration signal derived from (a) using local energy calculation. (c) LF/HF ratio. Hypopnea episode is shown by broken lines.

Third, two values obtained from the PSD are calculated: the low frequency (LF) power in the 0–0.1 Hz range and the high frequency power (HF) in the 0.2–0.33 Hz range. These two values represent the power of abnormal respiration rate and normal respiration rate respectively. The LF range was chosen according to the definition of hypopnea episodes durations (>10 s) since apnea and hypopnea events by definition last longer than 10 seconds [3], which is equivalent to a respiratory frequency inferior to 0.1 Hz. The HF range was chosen according to the general acceptation of normal respiration rates for adults [18]: 12–20 breaths/minute which is equivalent to 0.2–0.33 Hz. An example of LF and HF power is given in Figure 9. The value of LF increase when a hypopnea event occurs and the HF diminishes. In order to detect precisely the position of any hypopnea event we chose to use LF/HF ratio. The typical length of a hypopnea episode is 20–40 seconds [19], and then we chose to calculate LF/HF in windows of 30-s time. This operation is repeated every five seconds in order to detect the position in time of the hypopnea episode. A smaller time value (<5 s) would give better time resolution however will make the algorithm computations heavier. An example of the respiratory cycle variability (RCV) measure (in this paper the LF/HF ratio) obtained for the record 1 of the sleep dataset explained in Section 4.1 is shown in Figure 10.

## 4. Experiments

Nighttime sleep recording data acquired in our laboratory are used in this paper. Data is acquired using the cardiorespiratory belt sensor and the commercial system for ECG and respiration signals acquisition. Respiration signal in the case of commercial system was acquired using a TPG sensor attached to the nose. In the case of cardiorespiratory belt sensor, it was derived from the PVDF sensor output. The ECG signals are obtained from the cardiorespiratory belt sensor. In the following the dataset used in this paper is explained.

### 4.1. Sleep Dataset

This dataset contains data acquired during whole night sleeping. All data were acquired in our laboratory in a separate room equipped with a bed and air conditioning. Three recordings taken from this dataset are used in this paper for illustration, all belonging to the same male, aged 27 years. This subject’s data were chosen because they contain apnea or hypopnea episodes. The records are:

Record 1: 5 min episode taken from signals acquired in a supine position during sleep. The sampling frequency was set to 500 Hz and the total length of the record is 25,195 s. This record contains episode of hypopnea in the range 146–166 s.
Record 2: 5 min episode taken from signal acquired in a supine position during sleep. The sampling frequency was set to 500 Hz and the total length of the record is 25,189 s. This record contains episode of hypopnea in the range 129–151 s.
Record 3: 5 min episode taken from signals acquired in a supine position during sleep. The sampling frequency was set to 500 Hz and the total length of the record is 25,200 s. This record contains 2 episodes of hypopnea in the range 144–156 s and in the range 182–198 s. This record contains also an abnormal long respiration pattern in the range 52–61 s.

All records were resampled at 360 Hz to satisfy RC and QRS detection algorithm’s requirements.

## 5. Results

**Figure 11.**
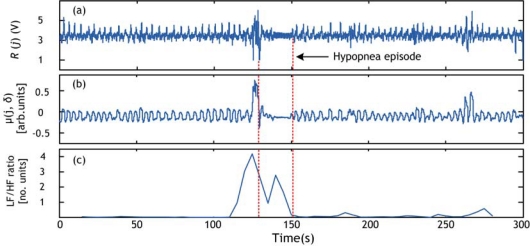
Hypopnea detection using respiratory cycle variability (RCV) for record 2. (a) PVDF sensor output. (b) Respiration signal derived from (a) using local energy calculation. (c) LF/HF ratio. Hypopnea episode is shown by broken lines.

**Figure 12.**
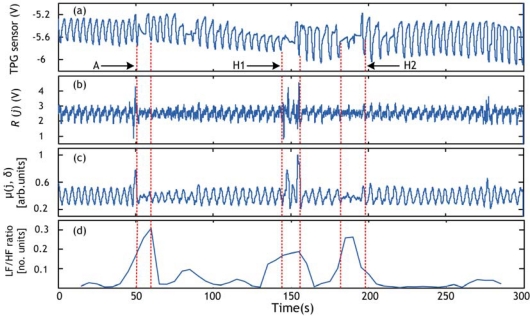
Hypopnea detection using respiratory cycle variability (RCV) for record 3. (a) Respiration signal obtained with commercial TPG sensor. (b) PVDF sensor output. (c) Respiration signal derived from (b) using local energy calculation. (d) LF/HF ratio. Hypopnea episode and abnormal respiration pattern are shown by broken lines. “H1” and “H2” represent hypopnea episodes whereas “A” represents abnormal respiration pattern.

The apnea/hypopnea method is applied to the sleep dataset, explained in Section 4.1, which contains episodes of hypopnea. The obtained results are validated by the presence of a clear event (peak) in the RCV curve. Examples of hypopnea episodes detected by this method are shown in Figures 10–12.

## 6. Discussion

An integrated sensor system was built and tested in our laboratory for cardiorespiratory signal acquisition. An application to the aforementioned system for health-related events detection, in particular apnea/hypopnea detection, was developed. This new method uses the respiratory PSD measures as detector of apnea/hypopnea events. Its principle is the use of the plot of the variations of the LF/HF ratio versus time to detect abnormal respiration patterns. The method was implemented in Matlab 7.1 and it was tested on some data samples taken from a sleep dataset acquired using the integrated sensor system. Results show that it is possible to detect apnea and hypopnea episodes using signals obtained from our cardiorespiratory belt sensor.

All the episodes of hypopnea present in records 1, 2 and 3 were detected. The method is quite insensitive to noise present in the time domain since its principle is calculating the PSD of the respiration signal. We should note that this method is very easy to implement and compute and gives online results, which can be very beneficial to physicians and health professionals. In addition, this method is based on processing simple signal (respiration) that makes it an easy tool for health diagnosis. The only parameter that needs to be fixed is the window parameter δ. For estimating respiration signal from PVDF sensor output or any other respiration sensors (for instance TPG sensor) window size value is better to be chosen inferior to typical respiration period, i.e., window length≤4 seconds: then, δ ≤ 2.

**Figure 13.**
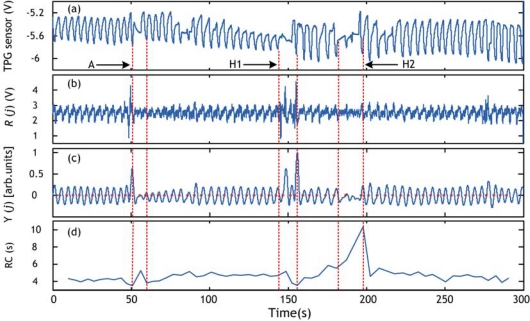
Hypopnea detection using respiratory cycle variability (RCV) for record 3. (a) Respiration signal obtained with commercial TPG sensor. (b) PVDF sensor output. (c) Respiration signal derived from (b) using local energy calculation and the RC detection algorithm. (d) RC calculated from (c). Circles represent respiratory cycle’s information estimated during inhalation time (positive slope). “H1” and “H2” represent hypopnea episodes whereas “A” represents abnormal respiration pattern.

As an illustration of the proposed method usefulness we show an example of hypopnea and abnormal respiration patterns in record 3 detected by an alternative method in which the successive respiratory cycle (RC) values are plotted versus time. We can see when comparing Figure 12 and Figure 13 that all events were detected using the LF/HF ratio marker (Figure 12d) whereas just one event (the hypopnea in the 182–198 s range) was detected by the respiratory cycle (RC) method in Figure 13d. This can be explained by the fact that the estimation of RC information is subject to the noise present in the respiratory signal. The RC information estimated wrongly will automatically lead to false detection. Conversely, our method using LF/HF ratio is immunized against such noise. The evidence is given by the accurate detection.

## 7. Conclusions

In this paper the measurement and analysis of cardiorespiratory signals were explained in details. The obtained results are summarized as follows:

1. The cardiorespiratory belt sensor system made in our laboratory proved able to detect cardiorespiratory information reliably.
2. A QRS detection algorithm and respiratory cycle algorithms were designed for the cardiorespiratory belt sensor. The QRS complex and the respiratory cycle information are accurately calculated.
3. A simple and efficient method for apnea and hypopnea detection was developed. The method is based on the calculation of low frequency (LF) to high frequency (HF) ratio value obtained from the respiration PSD. In this method the window parameter δ is an important parameter which has to be set judiciously in order to have an efficient detector. Results showed that the hypopnea events were detected accurately.
4. The system developed in our laboratory including integrated sensors and signal processing packages proved to be reliable in the detection of health-related events and it is well suited for the online analysis of long time series data and the detection of abrupt changes in physiological data.
